# On Injections in the Treatment of Uterine Diseases

**Published:** 1863-04

**Authors:** Robert Ellis

**Affiliations:** Obstetric Surgeon to the Chelsea and Belgrave Dispensary


### ON INJECTIONS IN THE TREATMENT OF UTERINE DISEASES.

By Robert Ellis, Esq., Obstetric Surgeon to the Chelsea and Belgrave Dispensary.

[The uses of injections may be learned by their failures. They are insufficient for the cure of ulceration if it has existed some time, and there is no satisfactory evidence that they can cure this condition effectually under any circumstances. In the great majority of cases they are even insufficient to cure leucorrhœa. They are useless for the cure of inflammatory induration and hypertrophy of the cervix, and they are equally ineffectual in the sole treatment of the spongy, indolent, patulous, and ulcerated cervix occasionally met with in obstetric practice.]

They are of great use in their secondary position as adjuvants to a higher class of remedies. For the relief of pain, for the removal of acrid discharges, for the deodorization of offensive, and for the suppression of exhausting fluxes, injections are of value. They are of use for giving tone to a relaxed and weakened organ, and as astringents for the support of the womb having a tendency to prolapse. Conjoined with judicious and appropriate cauterization, they are of the greatest use in hastening the cure of the inflamed and ulcerated womb; and it is of common observation, that patients who are careful in the use of injections, (as in private practice,) get well very much quicker, and with less pain, than those who (as at public institutions) neglect this means. When the cure is complete, injections are still of much use, but it is most difficult to convince the patient on this point. In married life it ought to be easy to induce the patient to persist in this most heathful duty; yet the reverse is the fact.

The *substances* adapted for injections in commonest use are of the stimulant and astringent kind. Of these, notwithstanding the opinions and practice of others, I consider the sulphate of zinc the most unjustifiable. I think I have seen it the cause of much irritation and mischief, and it is difficult to believe that the constant use of so poisonous a substance over so large a surface of mucous membrane can be other than injurious. The nitrate of silver is another substance most unsuited for injection, yet very frequently ordered for use. The mucus of the vaginal canal instantly decomposes it if used in a weak injection, and if in a stronger form, the excoriation of the external parts, together with the mischief inflicted on the linen, hands, and utensils of the patient, preclude its repeated employment. I have made use of a variety of substances for this purpose, but as simplicity and economy are chiefly of consequence in a daily matter of this sort, the result arrived at is, that a solution of alum, either alone or in decoction of oak bark, is, after all, the best and most effective injection we can prescribe. A mixture of equal parts of tannin and alum forms a more elegant, but also less costly substance as an astringent. For the anodyne injections, solutions of belladonna and of opium are the only serviceable remedies, and to these may be added the liquor plumbi and hydrocyanic acid with occasional good effect. For emollients, milk-and-water, linseed-tea, barley-water, and thin starch or gruel, are very valuable. The injection of gases and vapors is a very uncomfortable proceeding, and is not always free from a certain amount of risk, but considerable relief may sometimes be thus obtained when other means are useless. Of those most valuable are the carbonic acid gas and the vapor of chloroform.

Lastly, of the *instruments* for injection. Gooch's bent pipe instrument is a cumbrous and dangerous apparatus, very apt to get filthy, and to inflict injury on the cervix. The glass "female syringe" is a most absurd contrivance for cleansing a canal so capacious as that for which it is intended. It is also often broken, and sometimes within the canal itself. The ordinary pump, with elastic tube, has the disadvantage of requiring the assistance of a second person for its use. For the use of the poorer classes a simple and excellent instrument was contrived by me some years ago; it consists of a piece of gutta percha tube, five feet long, fitted at its upper end with an inch or two of elastic tubing; this could be slipped over the mouth of a common kettle, and the other end being placed in its proper position, the inversion of the kettle produced a constant stream of water of sufficient force to well wash out the canal. The same object may be also accomplished (and this method is lately used in France) by the use of a long syphon, the upper end being immersed in a reservoir of water, and the lower end retained in the canal by the patient. The French have an extraordinary variety of instruments for this purpose, amongst the most useful of which is one on the principle of the moderator lamp. Without exception, however, the most commodious and useful of all instruments for uterine injections is the elegant arrangement known as Dr. Kennedy's, and now becoming much used in this country. It may be employed either for gases or for fluids; as a douche or as an enema. An ingenious contrivance, known as the barrel syringe, made of caoutchouc, is also useful for this purpose; but the action of its valves is less to be relied upon than in the former instrument. For general use the douche just named is the best of all the varied forms of instruments for vaginal injections, and it will probably ultimately replace every other kind. Its valves require occasionally a little looking after and cleansing, but this is simple enough, as they merely consist of two metallic peas.—*Lancet, May* 31, 1862, *p.* 570.

